# Baclofen overdose: A curious case of medical sales representative

**DOI:** 10.1002/ccr3.6230

**Published:** 2022-08-09

**Authors:** Swotantra Gautam, Aakash Neupane, Mandita Chamlagain, Sandip Pokhrel, Durga Neupane, Rochana Acharya, Sagar Panthi

**Affiliations:** ^1^ PGY‐1, Resident, Internal Medicine Advent Health Orlando Florida USA; ^2^ B.P. Koirala Institute of Health Sciences Dharan Nepal; ^3^ Nidan Hospital Lalitpur Nepal

**Keywords:** altered sensorium, baclofen, metabolic alkalosis, overdose

## Abstract

In this case report, a medical sales representative consumed 250 mg of Baclofen out of curiosity. Baclofen has life‐threatening complications like seizures, respiratory depression, and coma. A majority of patients recover on symptomatic treatment. Baclofen has a great potential for abuse and overdose; therefore, its use must be strictly monitored.

## Abstract 2

Baclofen, commonly used muscle relaxant, carries great risk of abuse and poisoning. Baclofen overdose may cause coma, respiratory depression, and cardiovascular abnormalities in severe cases. The management is mainly symptomatic in an ICU setup with respiratory support. The interventions are tailored to each case as specific antidote is not available.

## 1 BACKGROUND

Baclofen, a synthetic derivative of the naturally occurring inhibitory neurotransmitter γ ‐aminobutyric acid (GABA), is a widely used muscle relaxant.^1^ It is used clinically to reduce flexor spasms and tone in conditions such as multiple sclerosis and spinal cord lesions.^2^ Although the exact mechanism of action is not well established, it is said to act principally on the GABA‐B receptor at the spinal level in therapeutic doses. It is also available as intrathecal preparation for long‐term indications such as severe spasticity, multiple sclerosis, or spasticity of cerebral origin.^3^

It has been increasingly used off‐label for the management of several disorders, including musculoskeletal pain, gastroesophageal reflux disease, and alcohol use disorder.

Baclofen overdose produces effects of physiologic depression resulting in bradycardia, decreased pulse, depressed neuromuscular activity, cardiac arrhythmias, and conduction defects.^4^ Effects of baclofen overdose are well defined and include coma, respiratory depression, seizures, and cardiac conduction abnormalities.^5^

## 2 CASE PRESENTATION

A 25‐year‐old male, with no prior comorbidities and no prior history of substance abuse, was brought to the Emergency Department (ED) of a tertiary hospital with an alleged history of ingestion of 10 tablets of Baclofen 25 mg each (total 250 mg) according to the evidence presented by the relatives in the ED in the form of empty strips of Baclofen at the bedside. The patient was reportedly found in a state of altered level of consciousness with frothing around the mouth and urinary incontinence, evident by bedwetting. One interesting thing to note is that patient is a medical representative involved in the marketing of baclofen itself and later admitted to have consumed the drug out of curiosity. No history of abnormal body movements, fever, or loss of consciousness was given. There was no significant medical, psychiatric, or substance abuse history. On examination in the ED, the patient was afebrile, pulse rate was 63 beats per minute, and respiratory rate 20 breaths per minute, blood pressure 110/80 mm of Hg, Spo2 94% at room air and Sp02 98% on 2 liters of oxygen supplementation, GCS 10/15 (E2V4M4). Pupils were 3 mm bilaterally and sluggish in response to light. Reflex was intact, posturing was not specific, cardiovascular system, respiratory system, and abdominal examination were normal.

On investigation, Arterial Blood Gas analysis (ABG) showed findings suggestive of metabolic alkalosis with pH of 7.255, HCO_3_ of 17.2 (reference range 22–26 meq/L), pCO_2_ 40 mm/Hg, and pO_2_ 111 mm of hg. Differential count showed raised neutrophil counts 87%, lymphocytes 13% (reference range: Neutrophil 55%–70%, lymphocytes 20%–40%). Urine routine/microscopy‐normal, urine toxicology screen was negative (Cocaine, Morphine, Amphetamine, Barbiturates, Benzodiazepines, Marijuana). A blood test for Acetaminophen was done for co‐ingestion. However, it was normal. Renal function test and liver function test were not significant. ECG showed normal sinus rhythm and CT head showed no abnormality. EEG was not done.

Symptomatic treatment was commenced in the emergency room as soon as the arrival of a patient after a quick initial assessment while investigations were carried out simultaneously. It included fluid support with normal saline and bicarbonate supplement, Inj. Levetiracetam 1 gm intravenously, Inj. Midazolam 1 mg intravenously, Inj. Ondansetron 4 mg iv, and Rabeprazole 20 mg IV. After initial treatment, the patient did not show any signs of improvement and the patient was transferred to another private health facility anticipating the need for an ICU. The patient was then transferred to the Intensive Care Unit (ICU) and monitored for seizures, bradycardia, and respiratory depression. The patient did not develop any seizures and bradycardia; however, the patient was aggressive during Foleys' catheterization. Atropine was kept ready at the bedside to react quickly in the event of bradycardia. However, atropine was not required. Intubation was not required. Respiration was later maintained with 2 L O_2_ via nasal prongs, spo2: 98%.

ICU Stay: Patient had improved consciousness, GCS:15/15 after 24 hours of ICU stay. He was not oriented to time initially; however, he later gave a history of taking 10 tablets of Baclofen (25 mg/1tab) out of curiosity about taste and its effect on the body. He was alert, and well oriented later. Details at the time of discharge were: BP:110/70, RR:20 SPO2:97% in room air, Temp:36 C HR:70, GCS: 15/15, shy and embarrassed. Total stay in hospital was for 2 days and he was discharged in DOPR (Discharged on Persistent Request). The patient was discharged on some oral antibiotics to complete the dosing, PPIs, and anti‐emetics. The patient was counseled to follow up in 2 weeks then after every month to monitor any residual side effects.

## 3 DISCUSSION

Initially, Baclofen was used primarily for its muscle relaxant properties. But, increasingly it is being used as an anti‐craving drug for those trying to quit smoking.^6^ Due to its structural homology with gamma‐hydroxybutyrate (GHB), it produces similar effects of coma, respiratory depression, seizures, and cardiac conduction abnormalities when taken in overdose. Thus, it is also being abused as party drugs, and rape drugs to produce unconsciousness and retrograde amnesia in unsuspecting victims. Our patient presented with a unique risk factor for ingestion. His job as a medical sales representative gave him access to a large supply of drugs and a potential opportunity to abuse the drug. Recent trends suggest that the abuse of prescription drugs is increasing rapidly all over the globe owing to the rise of Internet markets, the perception among the youth of it as safe drugs and increasing acceptance of sedatives, painkillers, and anti‐anxiety medications in the society,^7^ and our case suggests medical sales representative must also be taken into account while formulating policy. Studies have shown that an overdose of prescription drugs is more fatal than illicit drugs.^8^

Our patient reportedly consumed 250 mg of Baclofen orally. The recommended therapeutic regimen for adults for oral administration for spasticity is 5 mg orally three times a day; the amount may be increased up to 20 mg per dose three times a day (maximum dose, 80 mg per day in adults; 60 mg per day in children over 8 years of age). The safety and efficacy are not established for ages below 12 years. There appears to be a dose‐related effect whereby over‐doses of 200 mg or more are more likely to cause CNS depression or delirium, coma, and seizures and require ICU admission and longer hospital admissions. Due to unavailability of facilities, we could not perform assay of levels of baclofen in patients serum, which could have provided us a better picture of the level of toxicity.^9^

Our patient showed symptoms of altered sensorium in the form of altered level of consciousness, sedation, and sometimes aggressive behavior, especially during the intervention like Foley’s catheterization and drawing blood. Other possibilities of altered sensorium should be taken into consideration such as hypoxia, drug intoxication such as Phenobarbitol, Opioids, Ethanol, phenytoin, hypothermia, trauma, metabolic or endocrine disturbances, intracranial hemorrhage, and infections. We could not test for drug levels for phenobarbital, phenytoin, ethanol, acetaminophen, salicylate, or digoxin due to lack of facility and so we could not rule out possibility of their overdose. Imaging was not indicative of any signs of hemorrhage. The patient also presented with urinary incontinence which manifested in the form of wetting his clothes and bed. However, intubation was not needed as respiratory depression was not present. This may be due to the factor that he ingested around 250 mg only, which is close to the cutoff value currently described in the literature. A study done via a nationwide registry in Denmark demonstrated that serious effects of baclofen overdose started at ingestion of as low as a single dose of 150 mg of baclofen and required vigilant monitoring.^5^ Also, a synergistic effect was seen with alcohol and benzodiazepines when consumed together. Our patient presented no such risk factors. However, baclofen overdose, especially at higher doses, is potentially fatal. Several cases have been reported where baclofen overdose has mimicked brain death in adult as well as pediatric patients.^2^, ^10^, ^11^ Few cases have presented with unusual presentations such as catatonia and psychosis^12^ and anoxic encephalopathy.^13^

Baclofen overdose is associated with EEG changes as reported in some previous case studies. They reported a finding of burst suppression activity in EEG.^14^ Although quasi‐periodic generalized epileptiform discharges were also reported, it did not necessarily indicate the start of anti‐epileptic drugs.^15^ Unfortunately, in our case, EEG was not done.

Previous literature has suggested that most baclofen overdose patients recover on supportive management and only a few require hemodialysis, especially those with renal impairment.^11^, ^16^, ^17^ A case report shows a dose of baclofen as low as 10 mg can cause toxicity in patient with renal impairment. In such cases, immediate hemodialysis is the key.^18^ In a nationwide registry‐based survey, baclofen overdose mainly caused drowsiness, whereas very few cases went into coma and respiratory depression. The main approach to the treatment was also symptomatic.^19^ Our patient also recovered well on supportive management, and hemodialysis was not needed. Previous case studies have also documented similar outcomes. Even though patients presented with features mimicking brain death, they recovered within a week.^20^ Most recover within 72 hours.^2^ Atropine was found to be beneficial in a patient with respiratory and cardiac depression in a case study, indicating potential cholinergic action of baclofen toxicity.^21^ In our case, the patient did not show signs of bradycardia; hence, atropine administration was not required. This case report supports other previously published reports with regard to the requirement of vigilant supportive management along with intensive care unit backup in any case of baclofen poisoning for better outcome.

## 4 CONCLUSION

Baclofen, although not used widely in the context of Nepal, must be considered a potential source of abuse. It can produce toxicity in as low as 150 mg dose. Although complete recovery is seen in most cases, strict monitoring in an ICU setup is required to manage complications targeting respiratory depression and neurological complications.

## AUTHOR CONTRIBUTIONS

Swotantra Gautam: Involved in patient care, involved in literature review, manuscript writing, and proof reading. Aakash Neupane: Corresponding author, involved in literature review, manuscript writing, and proof reading. Mandita Chamlagain: Involved in patient care, involved in literature review, manuscript writing, and proof reading. Sandip Pokhrel: Involved in literature review, manuscript writing, and proof reading. Rochana Acharya: Involved in literature review, manuscript writing, and proof reading. Durga Neupane: Involved in literature review, manuscript writing, and proof reading. Sagar Panthi: Involved in literature review, manuscript writing, and proof reading.

## CONFLICT OF INTEREST

The authors declared no conflict of interest.

## CONSENT

Written informed consent was obtained from the patient to publish this report in accordance with the journal's patient consent policy.

## Data Availability

Data sharing is not applicable to this article as no new data were created or analyzed in this study.
